# Design of the Bimodal Grating Sensor with a Built-In Mode Demultiplexer

**DOI:** 10.3390/s23094327

**Published:** 2023-04-27

**Authors:** Andrei Tsarev

**Affiliations:** 1 Laboratory of Optical Materials and Structures, Rzhanov Institute of Semiconductor Physics, SB RAS, 630090 Novosibirsk, Russia; tsarev@isp.nsc.ru; Tel.: +7-913-4810-578; 2 Physics Department, Novosibirsk State University, 630090 Novosibirsk, Russia

**Keywords:** bimodal grating optical sensor, mode demultiplexer, silicon waveguide, numerical modeling

## Abstract

This new sensor design provides good volume sensitivity (around 1600 nm/RIU) via collinear diffraction by the asymmetric grating placed in the waveguide vicinity. It provides the mode transformation between the fundamental TE_0_ and the first TE_1_ modes of the silicon wire (0.22 μm by a 0.580 μm cross-section) in the water environment. In order to provide the wavelength interrogation with a better extinction ratio for the measuring signal, the grating design is incorporated with the mode filter/demultiplexer. It selects, by the compact directional coupler (maximum 4 μm wide and 14 μm long), only the first guided mode (close to the cutoff) and transmits it with small excess loss (about −0.5 dB) to the fundamental TE_0_ mode of the neighboring single mode silicon wire, having variable curvature and width ranging from 0.26 μm to 0.45 μm. At the same time, the parasitic crosstalk of the input TE_0_ mode is below −42 dB, and that provides the option of simple and accurate wavelength sensor interrogation. The environment index is measured by the spectral peak position of the transmitted TE_0_ mode power in the output single mode silicon wire waveguide of the directional coupler. This type of optical sensor is of high sensitivity (iLOD~ 2.1 × 10^−4^ RIU for taking into account the water absorption at 1550 nm) and could be manufactured by modern technology and a single-step etching process.

## 1. Introduction

Modern society uses multiple sensors and optical devices to make important contributions [1,2,3,4,5,6,7,8,9,10,11,12,13]. During the last decade, great interest, regarding optical sensors, was placed on the counterpart that utilizes bimodal interaction [13,14,15,16,17,18,19,20,21,22,23,24,25,26,27,28,29,30,31,32,33,34,35]. These sensors have a highly increased sensitivity (from one to several orders) in comparison with competitors using the same materials, but struggle to be realized in the traditional single mode design. These sensors can be roughly divided into three groups. The first two are based on the optical interference of two optical waves that propagate in two arms of the Mach Zender interferometer or as two different modes in the bimodal waveguide. The last variant uses the grating-assisted coupler and the collinear Bragg diffraction between two unidirectional optical modes. The common feature of all of these bimodal sensors is the fact that their sensitivity drastically increases inversely proportional to the group delay differences of the two modes, which takes part in the interaction. The conditions, when the group delay of two interaction optical waves is equal, are often called the “dispersion turning point” (DTP) [28,29] or “phase matching turning point” (PMTP) [30]. For the same optical length pass, the DTP condition is transformed to the case of the equal group indexes of two optical waves forming the filter part of the optical sensor.

Our attention is focused on the bimodal grating-assisted sensor on a silicon-on-insulator (SOI) structure, as it provides a unique opportunity for high sensitivity, mass production, and the possibility of multichannel measurement [35] by the wavelength interrogation method that measures several wavelength peaks corresponding to different detection materials by the single optical spectrum analyzer (OSA).

Typically, the bimodal optical sensors use the Bragg diffraction between the fundamental (TE_0_) and the first (TE_1_) guided modes. The sensor information, say, the environment index variation, is taken by measuring the wavelength position (wavelength drop) of the strong deep segment of the fundamental mode transmitted power. The parasitic power transmitted by the TE_1_ mode can be suppressed by a gradual change in the waveguide shape, say, by narrowing the waveguide width up to the single mode condition. It makes the TE_1_ mode radiate out of the waveguide pass and, thus, improves the quality of the measuring signal transmitted by the TE_0_ mode.

Unfortunately, in any case, the sensor parameters will be strongly influenced by the possible instability of the input power that is measured by OSA. In our understanding, the contribution of this factor can be significantly decreased if we make use of, for the wavelength interrogation, the TE_1_ guided mode diffracted power. This wave is propagated along the same waveguide and, for this purpose, one can use the mode filter/demuliplexer. 

At present, a lot of different integrated optics mode filtering and demultiplexing devices have been already developed [36,37,38,39,40,41,42,43,44,45,46,47]. For our particular case, this device should be intended for the demultiplexer TE_1_ guided mode that is close to the cutoff. It should have a small excess loss for the TE_1_ mode and low crosstalk for the TE_0_ mode, simultaneously. In addition, it can be manufactured jointly with the sensor element by a single-step CMOS-compatible fabrication process [48,49,50,51]. We make a choice regarding the widely used compact demultiplexer based on a tapered directional coupler [36,41,42,43,44,45,46]. 

The aim of this article is to propose and numerically prove the viability of the new design of the optical sensor that incorporates the grating-assisted bimodal waveguide and mode filter/demultiplexer, and demonstrate that it is easy to fabricate using the existing CMOS fabrication facility.

## 2. Materials and Methods

### 2.1. Sensor Design

**Figure 1:**
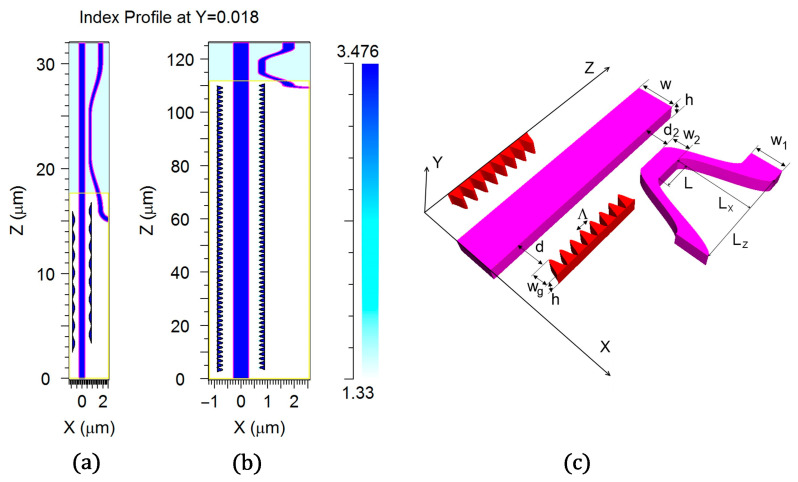
General design of the bimodal grating sensor element with the built-in mode filter based on the directional coupler. (**a**) Index profile cut for the small grating length (view with aspect ratio 1:1); (**b**) Index profile cut for the moderate grating length (view with aspect ratio 10:1); (**c**) 3D drawing of the device (aspect ratio 10:1) for the small grating length with the relevant optimized parameters: w = 0.58 µm, h = 0.22 µm m, w_1_ = 0.45 µm, w_2_ = 0.26 µm, d = 0.25–0.46 µm, w_g_ = 0.20 µm, d_2_ = 0.36 µm, ∧ = 1.63–1.65 µm, L = 4 µm, L_x_ = 1.1 µm, L_z_ = 14 µm.

For the realization of the aim to develop and study effective bimodal sensor design, we combined together the main features of the bimodal optical filter [52,53] and directional coupler demultiplexer [36,41,42,43,44,45,46] by making them accomplish the demonstration of a new feature. For the sensing part of the device, we used the optical filter design, but the segmented grating was replaced by the cosine-type arrangement that also had an antisymmetric position related to the waveguide axes (Figure 1). Both the waveguide and the grating were constructed by silicon, with an index of 3.476 at the optical wavelength of 1550 nm. The antisymmetric grating provided effective diffraction with the change of the mode number (TE_0_-TE_1_) and suppressed the diffraction without the change of the mode (TE_0_-TE_0_).

For the comparison of the environment sensor, two parameters are typically used; they are called volume sensitivity S_n_ and intrinsic Limits of Detection (iLoD) and are determined by the well-known formulas:

S_n_ = ∂λ/∂n_c_ = λ_0_·(∂N_0_/∂n_c_ − ∂N_1_/∂n_c_)/(N_0g_ − N_1g_) (1)

iLOD = δλ/S_n_, (2)

where N_i_ = N_i_(n_c_, λ_0_) and N_ig_ = N_ig_(n_c_, λ_0_) are the phase and group indexes of the fundamental (I = 0) and the first (I = 1) guided modes, respectively, n_c_—environment index, λ_0_—optical wavelength, and δλ—3 dB linewidth. The derivatives ∂N_i_/∂n_c_ of the mode indexes over the n_c_ variation depend on the optical waveguide structure.

The joint use of the multiplexer, consisting of a tapered coupler with two waveguides of varying width and two-mode SWG waveguides, was demonstrated as a bimodal sensor [33]. In this design, light was injected into the dual ports of a mode multiplexer and excitedthe TE_0_ and TE_1_ modes in the SWG waveguides. Their interference provided the twin dips in the transmitted power that was used for the wavelength interrogation. The gas sensitivity of 38.0 µm/RIU could be obtained with a sensing length of 1.9 mm.

A long-period grating with a sinusoidally modulated width of a silicon nitride rib waveguide was already demonstrated in the high-sensitivity bimodal sensor [34]. The interaction between the fundamental and the sixth mode provideda sensitivity reaching 11.5 µm/RIU, but more significant is that this sensitivity remained almost constant over a wide spectral range of at least 100 nm to around 1550 nm [34]. It is not typical of bimodal sensors having variable sensitivity to increase with the approaching optical wavelength up to the dispersion turning point. Unfortunately, this feature was reliable only for a rather small iLOD ~ 2.2 × 10^−3^ RIU.

The main reason for our using a cosine-type arrangement was that the long-period grating transformed the two-mode optical waveguide into the quasi-single mode waveguide as the grating coupler produced a small optical loss for the TE_0_ mode and a large loss for the TE_1_ mode. Similar results were described in a study of the strip and grating-loaded quasi-single mode waveguide on SOI [54]. It is important for us that this grating works as a sensor element, as well as producing a strong optical loss for the filtered TE_1_ mode and decreasing the power to be measured by the optical setup.

It was found that the cosine-type grating (see Figure 1) was better for this design than the segmented strip grating type as it provided an almost twice larger diffraction efficiency TE_0_-TE_1_ for a similar scattering loss of optical power in TE_1_ mode. In our design, the directional coupler of the demultiplexer was covered by SiO_2_ (index 1.444) and was placed just at the output of the grating-assisted bimodal waveguide surrounded by water (index 1.33). The coupler was formed by the input bimodal waveguide and the neighboring single mode waveguide with a variable width and shape, both of them being the cosine-like type (see Figure 1).

The simulations of the proposed structures were accomplished via direct three-dimensional (3D) modeling with the Finite Difference Time Domain (FDTD) method using the well-proved commercial software FullWave by Rsoft/Synopsis [55]. It should be noted that the 3D FDTD modeling needed a huge amount of computer resources (memory and simulation time). Thus, it is reliable only for the compact design of a grating with a small number of periods (typically 64). In addition, we used the symmetry design related to the plate Z = h/2 just crossing the center of the strip optical waveguide with height h and width w. Physically, it corresponded to the case of the suspended sensor that was surrounded by the liquid, and the directional coupler of the demultiplexer was surrounded by SiO_2_.

**Figure 2:**
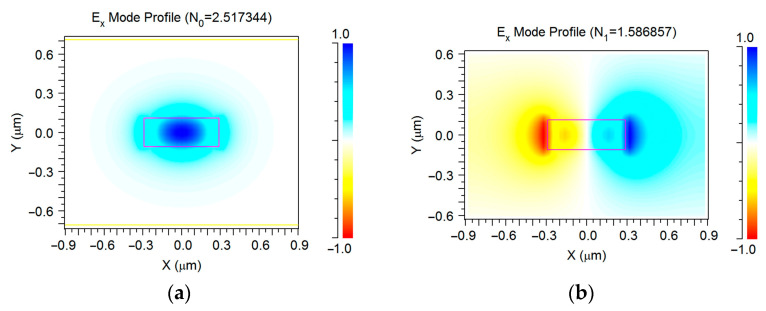

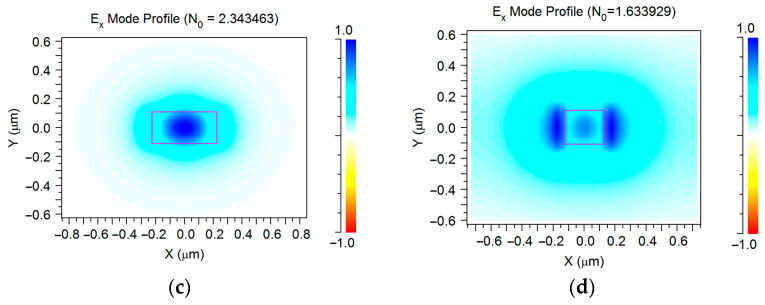
Real part of the mode profile of the main waveguide modes used in the interaction: (**a**) the TE_0_ mode in the basic optical waveguide with the 0.58 μm width; (**b**) the TE_1_ mode in the basic optical waveguide with the 0.58 μm width; (**c**) the TE_0_ mode in the right branch of optical waveguide with the 0.45 μm width on the output; (**d**) the TE_0_ mode in the right branch of optical waveguide with the 0.26 μm width used at coupler region. Simulation by 3D FEM.

This structure, built on SOI, supported the optical modes with the main profile distribution obtained by Finite Element Method (FEM), which is shown in Figure 2. The simulations were accomplished by the commercial software FemSIM by Rsoft/Synopsis [55]. Note that the TE_1_ mode had a much stronger penetration depth into the environment than that of the TE_0_, which provided higher dependence of an effective mode index N_i_ on the environment index n_c_. For example, we have collected the following data that are typical of the suspended silicon waveguide, as well as the fundamental and first modes that are not far from and close to a cutoff condition, respectively, ∂N_0_/∂n_c_ = 0.185 and ∂N_1_/∂n_c_ = 0.887. Thus, the bimodal sensor would provide high volume sensitivity (see (1)) for all optical wavelengths in the vicinity of a dispersion turning point, where N_0g_ = N_1g_.

The wavelength drop could be found by measuring the transmitting power by determining the spectrum tips (either TE_0_ mode minimum or TE_1_ mode maximum). For the first case, the easier way of measuring was a gradual decrease of the waveguide width to the single mode condition and then leaving the TE_1_ mode to be radiated out of the structure Thus, the measured transmitted power through the waveguide just corresponded to the TE_0_ mode.

The second variant was to filter the TE_1_ mode by the external element and measure its power. The mode filtering or demultiplexing belonged to a very good research task [36,37,38,39,40,41,42,43,44,45,46,47]. The demultiplexer based on the asymmetric Y-junction [37] had strong limitations for our use as it provided a rather small passband and low crosstalk that are out of our demand, even for the numerically obtained results. The experimental realization proved this conclusion and, anyway, a wavelength response contains large ripples in the transmitted power that were principally forbidden for our design as they randomly shift the measured wavelength drop position.

Better performances were obtained for the demultiplexer that combines a symmetric Y-junction and a multimode interference (MMI) waveguide [38]. The numerical simulation showed that the two-mode demultiplexer had a crosstalk of lower than −22 dB (from 1500 nm to 1600 nm) and crosstalk was reduced to less than −31 dB over the whole C band (1530−1560 nm). Taking into account the good fabrication tolerance of this element, it could be a good candidate for our sensor implementation, but it is desirable to have a better crosstalk and larger bandwidth.

The alternative design used two MMI waveguides [39,40] that traditionally belonged to the most fabrication-tolerant optical elements. To provide a mode filtering, it contained an additional mode shifting element. All of these made the device rather long, although it had a good tolerance to fabrication errors (±50 nm). This device hadan excess loss of 0.15 dB at a wavelength of 1550 nm, and <1 dB over the wavelength range of 1520–1580 nm. The crosstalk was less than −20 dB for a bandwidth of 60 nm. That was also very good, but not enough for our requirements. Thus, we chose the directional coupler variant for the two-mode demultiplexer.

The directional coupler mode filter/demultiplexer could be compact, easy to manufacture, and provide a small excess loss and low crosstalk, simultaneously. This type of optical element was under intensive study during the last few years [36,41,42,43,44,45,46]. It used different designs: the no-taper structure, one-taper structures, and two-taper structure. The last of them could eliminate the mode mismatch and reach a high performance of low excess loss (≤0.5 dB) and low crosstalk (≥20 dB) in a wide wavelength range (≥200 nm). However, in order to obtain a high conversion efficiency that is close to 100%, the tapered length was made to be big enough (400 μm) [45].

The original mode multiplexing realized just on the fiber output was experimentally demonstrated [47] by the grating coupler that accomplished direct transformation from the guided TE_1_ mode to the fiber LP_11_ mode. This variant was compared with the alternative as an asymmetrical directional coupler [46] in order to transform the TE_0_ into the TE_1_-guided mode. The limitation of this design was in the limited grating coupler bandwidth.

We studied a compact variant of the asymmetric directional coupler with extra high characteristics that suited our requirements for the bimodal grating sensor. In order to provide a large optical bandwidth, we designed and optimized the asymmetric directional coupler that hadthe small coupling length (4 μm) and short (5 μm) curved parts that were needed to decouple the demultiplex input/output waveguides from the main bimodal waveguide (Figure 1). It must be noted that the best efficiency corresponded to the unusual conditions when the guided waves in the two coupled waveguides had slightly different mode indexes (see Figure 2). Nevertheless, at the optimal variation of output waveguide shape and position, it provided almost a 90% efficiency of transformation between the TE_1_ and TE_0_ guided mode of two coupled waveguides (see below).

**Figure 3:**
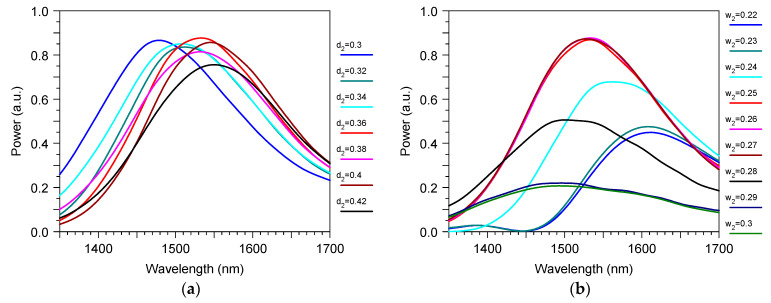
The wavelength spectrum of the transmitted power through the mode filter based on the directional coupler: (**a**) as a function of the directional coupler gap d_2_ (μm); (**b**) as a function of the minimum waveguide width w_2_ (μm) of the right arm of the directional coupler. Input is the TE_1_ mode in the left branch of the waveguide and Output is the TE_0_ mode in the right branch of the waveguide. Simulation by 3D FDTD.

**Figure 4:**
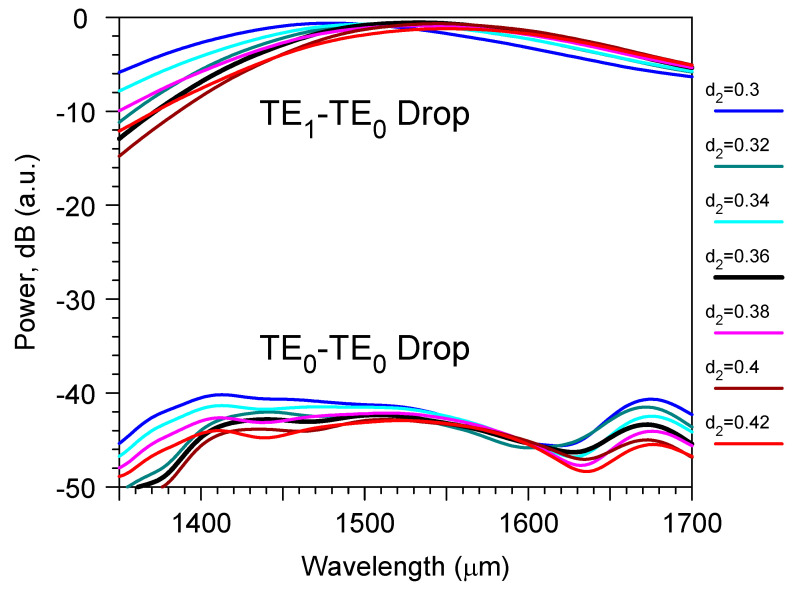
The wavelength spectrum (on the dB scale) of the transmitted power through the mode filter based on the directional coupler for different gaps d_2_ (μm) of the directional coupler. Inputs are the TE_0_ or TE_1_ modes in the left branch of the waveguide and Output is the TE_0_ mode in the right branch of the waveguide Simulation by 3D FDTD.

The results of the optimization procedure are shown in Figure 3. It was accomplished by direct 3D FDTD modeling and determined that, at an optical wavelength 1.53 μm, the optimal gap and minimum width of the curve arm of the directional coupler were 0.36 μm and 0.26 μm, respectively. In optimal conditions (Figure 1) the TE_1_ mode from the main two-mode waveguide transformed into the TE_0_ mode of the output single-mode waveguide with about a −0.5 dB power penalty. At the same time, crosstalk from the input TE_0_ mode to the output TE_0_ mode was below −42 dB, and the demultiplexer −3 dB passband was about 214 nm (Figure 4). The −1 dB passband was also very large and it was about 122 nm.

### 2.2. Grating Sensor Element

The grating sensor element design was more complicated than that of the mode demultiplexer. It was known that the collinear diffraction efficiency TE_0_-TE_1_ is determined by the interacting modes overlapping with the index perturbation formed by the grating. At the same time, the radiation loss is also dependent on the TE_1_ mode overlapping with the grating perturbation. Thus, any variation in the grating design caused changes both in the efficiency of diffraction TE_0_-TE_1_ and the radiation loss of the TE_1_ mode.

**Figure 5:**
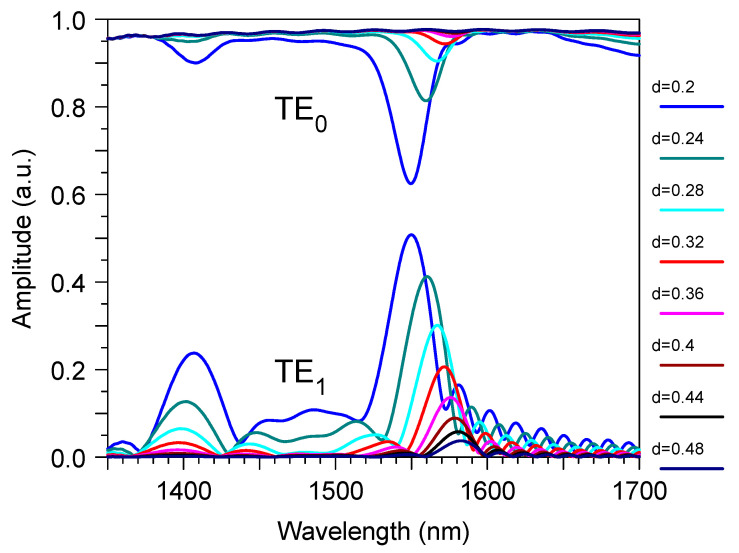
The wavelength spectrum for the amplitude of the transmitted power through the bimodal grating sensor element for the different spacing d (μm) between the waveguide and grating. Simulation by 3D FDTD.

It resulted in the fact, that for the cases of high diffraction efficiency, for example, for a long grating or a small gap between the grating and the waveguide, the power loss may have exceeded the diffraction efficiency. For example, from the data plotted in Figure 5, one can find that, for the gap of 0.20 μm, the TE_0_ mode power suppression was very strong (70%), but only 27% of it stayed within the waveguide, and most (43%) of it was lost by the radiation.

At the same time, by increasing the gap, the ratio loss/efficiency was decreased and became reasonable for practical application. Say, for the gap of 0.24 μm, we had a 30% TE_0_ power suppression making most of it (18%) correspond to the TE_1_ transmitted power and only 12%—for the radiating loss.

At the same time, by using the sensor with the alternative segmented grating [52,53] we will obtain worse results. For example, for the gap of 0.20 μm, we will obtain 61% of the TE_0_ mode power suppression that corresponds to the 18% efficiency of the diffracted TE_1_ mode, and a huge loss of about 42%, that is, 2.33 times the diffraction efficiency. A moderate loss of about 20% was obtained for the gap of 0.24 μm, which corresponded to a 33% suppression of the transmitted TE_0_ mode and 13% of the diffracted TE_1_ mode. Thus, the cosine-type grating design was about twice as good from the point of using the power of the diffracted wave generated during the bimodal interaction.

## 3. Results

**Figure 6:**
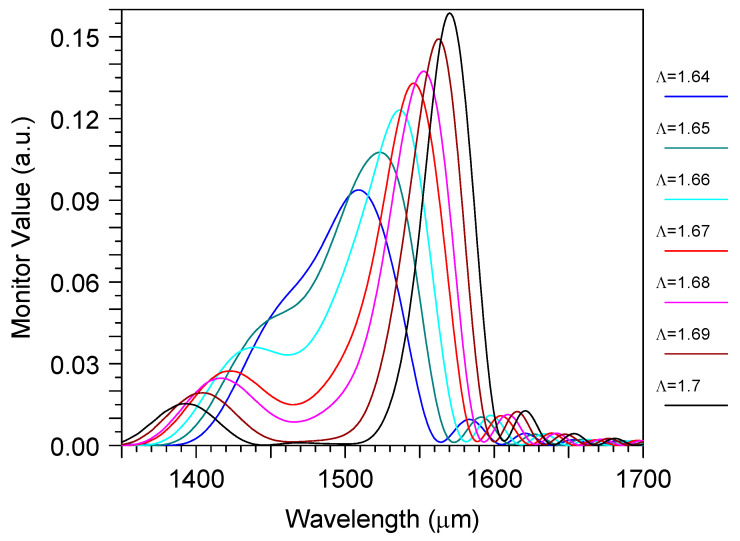
The wavelength spectrum of the transmitted power through the bimodal grating sensor element for the different grating periods ∧ (μm). Simulation by 3D FDTD.

The bimodal grating sensor parameters were the subject of optimization. For example, the operation optical wavelength dependedon the gap between the waveguide and the grating (Figure 5), as well as on the grating period ∧ (Figure 6). The increase of the last moved the operation wavelength to the dispersion tuning point and, thus, increased the sensitivity, according to Equation (1), at the decrease of the spectral bandwidth [27].

**Figure 7:**
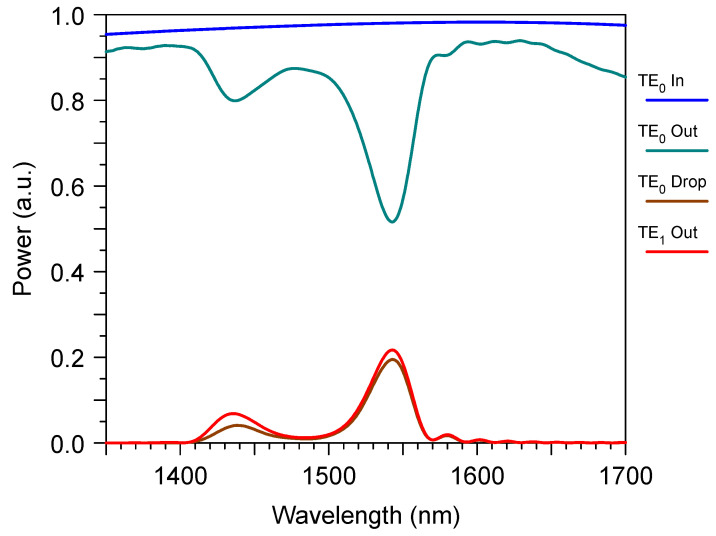
The wavelength spectrum of the transmitted power through the bimodal grating sensor element with the mode filter based on the directional coupler. “TE_0_ in”, “TE_0_ out” and “TE_1_ out” are the transmitted mode power at the input and the output of the sensor grating section; “TE_0_ Drop” is the transmitted mode power at the output of the right branch of the directional coupler. Simulation by 3D.

As it was mentioned before, our design incorporated both the grating sensor element and the mode demultiplexer. The typical signals that can be measured by OSA for this device are shown in Figure 7. According to the traditional design, one can measure the power “TE_0_ Out” at the end of the bimodal waveguide. According to our approach, it is better to measure the power “TE_1_ Drop” at the demultiplexer output. It is important that, for the proper design, this signal will be slightly (only by −0.5 dB) smaller than the optical power “TE_1_ Out” that is determined just at the end of the grating sensing element.

**Figure 8:**
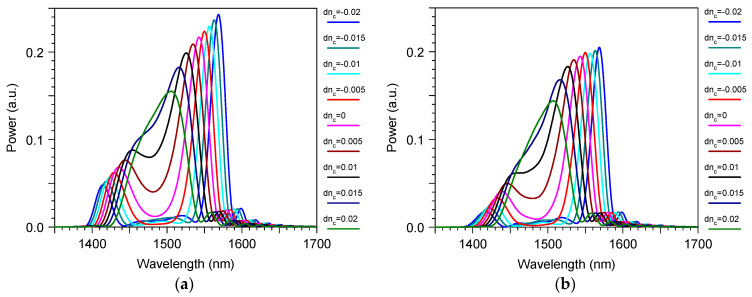
The wavelength spectrum for a different index increment in water for the transmitted power through the bimodal grating sensor element with the mode filter based on the directional coupler: (**a**) “TE_1_ out” for the power at the output of the grating section; (**b**) “TE_0_ Drop” for the power at the output of the directional coupler demultiplexer. Simulation by 3D FDTD.

The sensing properties are illustrated in Figure 8. One can see, with the water index perturbation by dn_c_, we hada monotonic shift in the optical wavelength drop. Note that, due to the wide working passband of the demultiplexer, the peak positions at its output followed the similar TE_1_ mode peaks produced by the grating coupler. At the same time, the additional peaks at the short spectrum wavelength range were strongly disturbed. They were out of the filter passband and, thus, were not used for our sensing purpose.

These results demonstrate the well-known feature of the bimodal sensor, that the sensitivity increases as the wavelength dropapproaches the dispersion tuning point (in our case, 1490 nm, which is just the middle position between the short and long wavelength peak of the spectrum curve). At the same time, regarding this, the approaching one also increased the 3 dB linewidth δλ that determined the intrinsic Limits of Detection (2).

**Figure 9:**
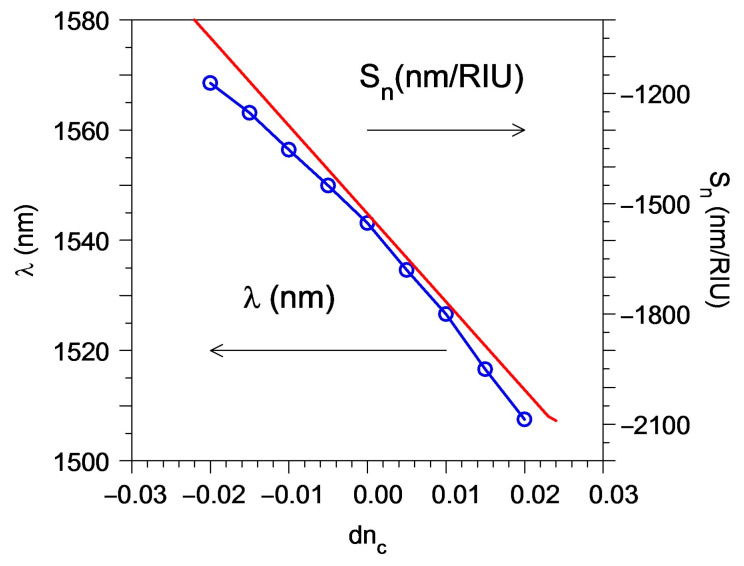
The dependence on the index increment in water for the wavelength drop λ (see blue line with dots) and sensitivity S_n_ (see red line) of the bimodal grating sensor element with the mode filter based on the directional coupler obtained by the “TE_0_ Drop” signal. Simulation by 3D FDTD.

Due to the memory limitation for the 3D FDTD simulation, our results were obtained only for a small number of grating periods (64), and that demonstrateda sensitivity of around 1600 nm/RIU, which varied in the range of ±500 nm/RIU for the index perturbation of ±0.02 RIU (Figure 9). For the smaller index perturbation range in the liquid, the sensitivity variation would also decrease.

The small grating length (only 108 μm) provided the small iLOD = 0.022 RIU. However, our results made it possible to estimate the real device parameters with a recommended length of around 1 cm. Say, if we fixed the position of the wavelength dropat the point when S_n_ = 1600 nm/RIU, then we would obtain a filter linewidth δλ~0.19 nm that corresponds to iLOD ~ 10^−4^ RIU for the ideal grating in the liquid without loss. As the water absorption at 1550 nm was rather high (9.6 cm^−1^), we hadan intrinsic limited linewidth [56] of about 0.28 nm and the real iLOD ~ 2.1 × 10^−4^ RIU, which is enough for many practical applications. This longer grating length used a large gap of around 0.46 μm (Figure 5) in order to keep the desired diffraction efficiency of ~20%. In addition, according to Figure 6, it also used the corrected grating period of 1.63 μm.

Note that it is possible to keep the same iLOD with a much larger sensitivity value by decreasing the period and by choosing the wavelength drop to be closer to the dispersion tuning point, but this will provide a higher inhomogeneous dependence of the sensitivity and, thus, the smaller operation range in the change of the liquid index that is measured by the sensor. For example, in the case that the sensor demonstrates a sensitivity of around 1770 nm/RIU, which varies at the range ± 240 nm/RIU for the index perturbation from 0 to 0.02 RIU (Figure 9).

It must be mentioned that this iLOD ~ 2.1 × 10^−4^ RIU was around the detection limit iLoD = 2.4 × 10^−4^ RIU of an ideal resonator sensor in water at an optical wavelength of 1550 nm. To obtain this value, an ideal Fabry-Perot cavity was considered and it was assumed that the light traveled entirely in the water, with no loss mechanism other than water absorption [56]. This means that it is of no sense to further increase the grating length of the bimodal water sensor at an optical wavelength of around 1550 nm as the resolution would be mainly limited by the water absorption [56]. However, it is known that a better iLOD ≤ 1 × 10^−4^ RIU could be obtained in water at an optical wavelength of 1350 nm due to the much smaller optical loss in this optical range [57].

This sensor design containeda structure with the same height as the input optical waveguide, the grating, and the directional coupler of the mode demultiplexer. The sensor parameters were rather stable to the variation of the main design features if they were controlled with an accuracy of around ± 10 nm. For example, it is demonstrated in Figure 3 and Figure 4 that the variation of mode filter/demultiplexer dimensions within this limit did not produce any significant change in the excess loss or crosstalk of this optical element. One can see the similar stability of the total sensor parameters in Figure 5 and in Figure 6 for the variation of the grating coupler features (gap d and period ∧). This means that the proposed device could be manufactured by the modern technology that has already developed for silicon photonics [48,49,50,51].

## 4. Conclusions

The direct numerical modeling by 3D FDTD proves the main conception of the modified bimodal grating sensor. The new device contains an asymmetric long wavelength grating of the cosine-type shape in the two-mode waveguide vicinity. The collinear diffraction of the TE_0_ mode produces the TE_1_ mode that is further selected by the adiabatic directional coupler demultiplexer that has an excess loss of around −0.5 dB, parasitic crosstalk of the TE_0_ mode of around −42 dB, and operation 3 dB passband of around 214 nm at the central wavelength of about 1530 nm.

The optimization of this type of optical sensor is accomplished by the numerical modeling of a short device and by extending the results for practically important structural dimensions. The main idea is to combine the bimodal optical filter and mode demultiplexer, combining the improved parameters together to meet the joint requirement of the proposed sensor element. A traditional bimodal grating filter utilizes the segmented periodic modulation of the media. It provides increased scattering optical loss of the diffracted TE_1_ mode. The implementation of the novel design of grating with the cosine-type shape decreases the scattering loss of the TE_1_ mode related to the segmented grating design twofold. The optimal efficiency for the TE_0_-TE_1_ diffraction is around 20%, in order to provide a small scattering loss that is important for this sensor design. Depending on the grating length, one has to control the spacing between the waveguide core and the grating. The grating strongly disturbs the optical waveguide properties; thus, for a different spacing, one has to use a different grating period in order to choose the appropriate optical wavelength related to the dispersion tuning point that controls the sensitivity according to Equation (1).

The proposed sensor is intended to measure the power of the diffracted TE_1_ mode that has to be filtered by the mode demultiplexer built by a directional coupler. The last one has to be rather compact (<15 µm), utilize a large optical band (>210 nm), have a small excess loss (~−0.5 dB), and have high crosstalk (<−40 dB). The compromise for the structure that better suits all of these requirements was found after the intensive numerical modeling by 3D FDTD. All of these understandings make up the background and the main results of the current paper.

For the compact device (126 μm by 4 μm) that used a short grating with 64 periods, we have a sensitivity ranging from −1100 nm/RIU to −2100 nm/RIU for the water index perturbation ±0.02. The short device provides a small iLOD = 0.022 RIU. However, it can be proven that, for the practical 1 cm long device, one can obtain iLOD ~ 2.1 × 10^−4^ RIU or iLOD ~ 10^−4^ RIU at an optical wavelength of 1550 nm or 1350 nm, respectively. That is useful for many practical applications. The large sensitivity will provide a detecting limit of about 10^−5^ RIU for an OSA resolution of 0.01 nm. The same detection limit could be obtained using the OSA with a worse resolution by choosing the operation wavelength closer to the dispersion tuning point that drastically increases the sensitivity (up to 50 μm/RIU) [27] during the decrease of the measuring range of the water index perturbation.

This type of bimodal sensor can be developed via conventional CMOC technology using a single-step etching developing process. This is promising for the possibility that these kinds of optical sensors will be increasingly cheap in terms of their production, and their high sensitivity makes it possible to use them with the cheapest type of OSA arrangement.

## Data Availability

The data are unavailable due to privacy as the simulation is accomplished by the licensee of Rsoft/Synopsis.
